# Theoretical interpretation of drivers’ gaze strategy influenced by optical flow

**DOI:** 10.1038/s41598-021-82062-1

**Published:** 2021-01-27

**Authors:** Yuki Okafuji, Takanori Fukao

**Affiliations:** 1 School of Information Science and Engineering, Ritsumeikan University, Kusatsu, Shiga 525-8577 Japan; 2 Department of Mechano-Informatics, The University of Tokyo, Bunkyo-ku, Tokyo 113-8656 Japan

**Keywords:** Psychology, Human behaviour

## Abstract

Driver analysis, particularly revealing where drivers gaze, is a key factor in understanding drivers’ perception. Several studies have examined drivers’ gaze behavior and the two main hypotheses that have been developed are Tangent Point (TP) and Future Path Point (FP). TP is a point on the inner side of the lane, where the driver’s gaze direction becomes tangential with the lane edge. FP is an arbitrary single point on the ideal future path for an individual driver on the road. The location of this single point is dependent on the individual driver. While these gaze points have been verified and discussed by various psychological experiments, it is unclear why drivers gaze at these points. Therefore, in this study, we used optical flow theory to understand drivers’ gaze strategy. Optical flow theory is a method to quantify the extent to which drivers can perceive the future path of the vehicle. The results of numerical simulations demonstrated that optical flow theory can potentially estimate drivers’ gaze behavior. We also conducted an experiment in which the observed driver gaze behavior was compared to calculated gaze strategy based on optical flow theory. The experimental results demonstrate that drivers’ gaze can be estimated with an accuracy of 70.8% and 65.1% on circular and straight paths, respectively. Thus, these results suggest that optical flow theory can be a determining factor in drivers’ gaze strategy.

## Introduction

Humans use their visual systems to guide their locomotor control; in the driving context, drivers take advantage of visual information to safely operate their vehicles^1,2^. The typical pattern of gaze in driving is characterized as the driver looking to where they want to go and steering towards that point^3,4^. Thus, revealing driver gaze point during driving is a key factor in a holistic understanding of driving behavior because driver gaze is the first sensory information process in driving.

**Figure 1:**
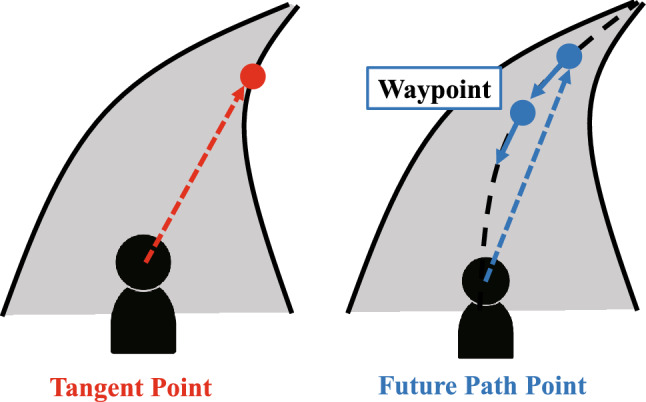
Difference between Tangent Point and Future Path Point. In the right figure, the dot line means an invisible desired path.

For decades, several studies have attempted to elucidate drivers’ visual characteristics and, as shown in Fig. 1^5,6^, there are two major hypotheses regarding guiding fixations directly related to steering performance: Tangent Point and Future Path Point. These gaze behaviors have been compared and discussed through both naturalistic and simulated driving tasks. The Tangent Point (TP) was first proposed as a type of drivers’ gaze behavior and has been reported in various studies^3,7–10^. The TP is a point on the inner side of the lane, where the driver’s gaze direction becomes tangential to the lane edge^3^. By gazing at the TP, the driver is said to have the advantage of estimating the road curvature geometrically, which provides useful information for steering^3^. The TP hypothesis thus assumes that steering performance can be improved by gazing at the TP and improvements to the smoothness and stability of steering have been observed through such experiments^8,9^. In contrast, the Future Path Point (FP) is an arbitrary single point on the ideal future path for an individual driver on the road; for instance, the center of the path or corner-cutting path^11^. This gaze behavior has also been observed in various studies^4,11–19^. The TP is a behavioral concept in which the driver’s gaze point is uniquely determined by the environment and vehicle condition; in other words, the individual characteristics of the driver are not included. In contrast, the gaze distance (or preview distance) in the FP gaze concept varies according to the individual driver; thus, the drivers gaze at a single point on the ideal future path corresponding to their gaze distance. If drivers gaze at the TP during driving, the vehicle runs towards the inner edge, which gives unstable control because the drivers unconsciously tend to steer towards their gaze point^13,14^. Therefore, FP gaze behavior can be considered a more natural way of maintaining stable control. In addition, a detailed analysis of eye movements during driving suggests that drivers gaze at the FP^17–19^. Meanwhile, some studies suggest that the TP or FP vary with the road environment and vehicle conditions, rather than being uniquely determined by either type of gaze behavior^20,21^. Similarly, TP and FP gaze behavior have been observed not only in driving but also in cyclists^22^ and motorcycle riders^23^. From these various results of previous studies, drivers’ gaze behavior has largely been clarified; however, the reason behind such gaze behavior, that is, why drivers gaze at a particular point during driving, is not yet fully understood. Understanding the reason for gaze behavior is a key factor in understanding drivers’ perceptions more holistically.

**Figure 2:**
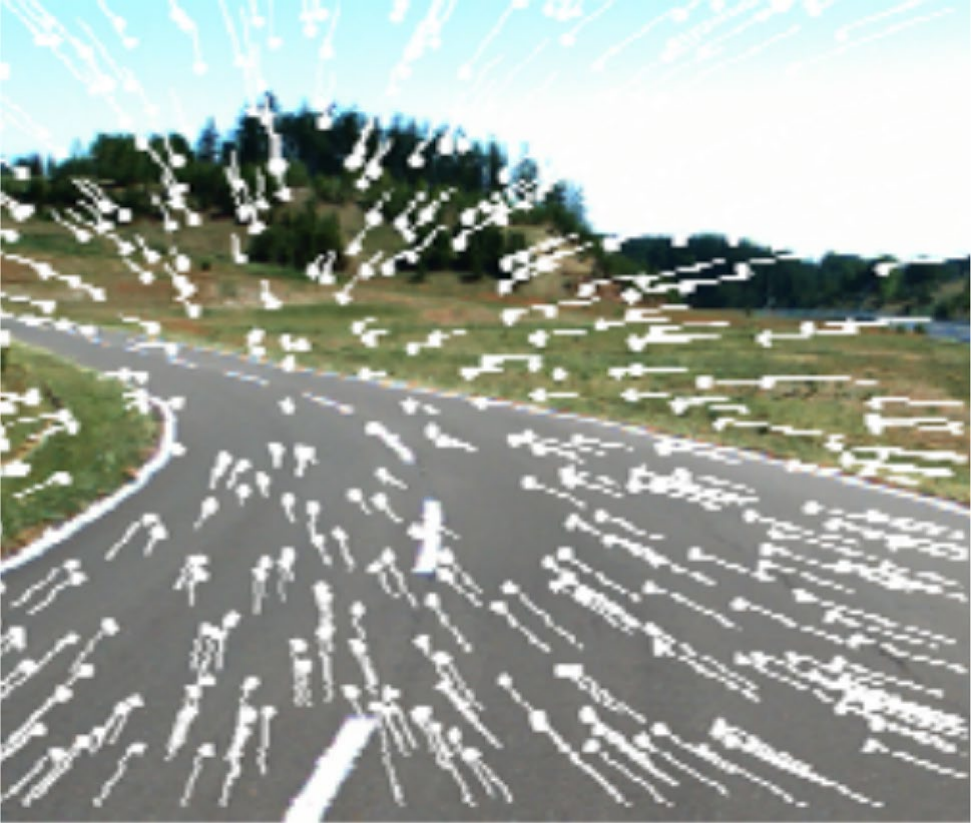
Optical flow generated by surrounding environment.

The visual cue required for driving itself, particularly for steering control, is said to be the geometrical road information and optical flow^24,25^. Optical flow is defined as the velocity vector generated by an observer’s motion through a surrounding environment and reflected in the observer’s visual field, as shown in Fig. 2^26^. Optical flow can reflect observers’ translation and rotation movements. Humans perceive the direction of self-motion from optical flow in order to accurately guide locomotor control^27^. In addition, it has also been demonstrated that drivers preferentially acquire information, comprising road information or optical flow, according to necessary control based on the current vehicle state^28^. For example, drivers tend to gaze at the road edge when lane-keeping is strongly required, whereas they gaze at a more distant point where they can easily perceive the optical flow when the vehicle control is stable. Accordingly, we assume that drivers’ gaze strategy may also be determined by the need to acquire the information required for steering control. Our hypothesis is that the driver actively performs effective gaze behavior to perceive the accurate direction of self-motion in order for accurate steering control. Incidentally, Vansteenkiste et al. reported that cyclists’ gaze strategy is often different from drivers’ gaze behavior^22^, due to the difference in optical flow speed or task demands. Thus, in this study, we assume that drivers’ gaze behavior can be interpreted by optical flow, which is one aspect of the information required for driving.

The aim of this study is to interpret drivers’ gaze behavior (TP or FP fixation) from the perspective of optical flow. From the results of previous studies, FP fixation is a more natural drivers’ gaze strategy, but we consider that such gaze strategy depends on the environmental situation and vehicle state; thus, more important than knowing where drivers gaze is knowing why they gaze at the FP or TP while driving. In this study, we hypothesized that drivers’ gaze behavior is determined by the extent to which they can perceive the predicted future path of the vehicle since the optical flow, which can reflect the direction of self-motion, might influence drivers’ gaze behavior. If the perception of the accurate direction of self-motion is necessary for accurate steering control, then drivers might perform gaze behavior to perceive the accurate direction of self-motion. The contribution of this study is, first, to show the possibility that optical flow theory can explain drivers’ gaze behavior through numerical simulations. Furthermore, we compared the results between the calculated gaze strategy by optical flow theory and drivers’ gaze behavior observed from the experiment. Using the results of numerical simulations and experiments, we discussed whether optical flow theory can be interpreted as the reason why drivers gaze at the FP or TP during driving.

This paper is mainly divided into three parts. In the first part, we derive optical flow theory that is a method to quantify the extent to which drivers can perceive the future path of the vehicle. In addition, we show a way of how we can evaluate driver gaze behavior by optical flow theory. In the second part, we demonstrate the theoretical drivers’ gaze strategy by optical flow theory in the numerical simulations. These results are compared to the results in the literature to suggest the potential for drivers’ gaze strategy to be interpreted as optical flow theory. Finally, the results in the experiment to compare the difference between the observed drivers’ gaze behavior and calculated gaze strategy by optical flow theory are discussed.

The preliminary research for this study was presented at a conference proceedings^29^, where we reported the fundamental theoretical idea and simulation results. The current paper has been refined accordingly, and also provides a comparison of the results from theoretical and experimental gaze behavior.

## Modeling of optical flow

It is well known that optical flow information itself gives the direction of self-motion from psychological experiments^30^. On the other hand, we use the theoretical optical flow in this study by referring to^31^. The theoretical optical flow is a model that the direction of self-motion can really be represented by optical flow in theory. The advantage of using this model is to quantify the extent to which drivers can perceive the future path of the vehicle.

**Figure 3:**
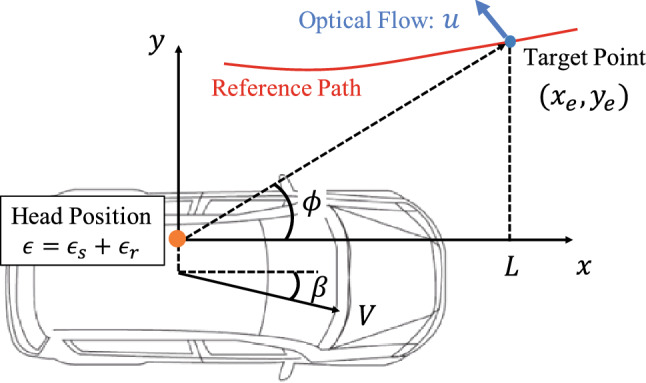
Vehicle coordinate. Optical flow affected by seat position and driver’s roll movement.

When we derive the theoretical optical flow, we assume that the driver is located in the driver’s seat position of the car, and that the driver’s head position has roll movement caused by vehicle motion. Optical flow perceived by the driver is considered to be a combination of the flow generated by the vehicle motion and the driver’s own motion in the vehicle. Therefore, we first built a mathematical model based on the vehicle chassis system; then, we defined optical flow in the visual field of drivers. In this study, optical flow is defined as the differentiation of the angle objects. Figure 3 shows the vehicle coordination for modeling optical flow. We defined $\epsilon$ as the lateral deviation between the driver’s head position and the center of the vehicle. This deviation can be divided into two parameters: the seat position $\epsilon _s$ and driver’s roll movement $\epsilon _r$. In this section, rolling, pitching, and vertical motions are negligible. Thus, the vehicle motion is constrained to the horizontal movement and optical flow is generated only on the ground and projected in the driver’s vision.

When a target point on a target path is expressed as $[x_e, y_e, z_e ]^T$, the horizontal optical flow *u* generated by the vehicle motion can be described as

1 $$\begin{aligned} u = \frac{d\phi }{dt} = \frac{x_e\dot{y}_e - \dot{x}_ey_e}{x_e^2+y_e^2}, \end{aligned}$$

where $\phi$ represents an angle between the vehicle direction and target point.

Next, we show the velocity at the target point viewed by the driver. We assume that the driver continuously gazes at an arbitrary point fixed on the ground during a certain time, which is sometimes called waypoint strategy. Then, the velocity is as follows:

2 $$\begin{aligned} \frac{d}{dt}\left[ \begin{array}{l} x_e\\ y_e\\ z_e \end{array}\right] = \left[ \begin{array}{c} -V \cos \beta \\ - V \sin \beta - {\dot{\epsilon }}_r \\ 0 \end{array} \right] + \left[ \begin{array}{c} 0 \\ 0 \\ - {\dot{\phi }}_{vp} \end{array} \right] \times \left[ \begin{array}{c} x_e\\ y_e + \epsilon \\ z_e \end{array} \right] , \end{aligned}$$

where *V* is the vehicle velocity, $\beta$ denotes the vehicle lateral slip angle, and ${\dot{\phi }}_{vp}$ represents the yaw rate combined with the vehicle yaw rate $\gamma$ and the driver’s eye movement ${\dot{\phi }}_p$.

To reflect the perception of the human driver, we need to consider the combined optical flow generated by the vehicle and the driver’s motion. To introduce eye movement, we need to consider one of eye movement during driving: Optokinetic Nystagmus (OKN)^32^. Drivers’ eyes are forcibly rolled by the influence of the optical flow generated on the ground. This behavior is called OKN. Therefore, when the driver is gazing at an intended target point while driving, their eye movement is affected only by the optical flow, not by their body motion. In this study, we simply describe OKN behavior as eye movement ${\dot{\phi }}_p$. The combined motion ${\dot{\phi }}_{vp}$ is given as

3 $$\begin{aligned} {\dot{\phi }}_{vp} = \gamma + {\dot{\beta }} + {\dot{\phi }}_p, \end{aligned}$$

where ${\dot{\phi }}_p$ is discussed later.

Using (1), (2), and (3), we can obtain the optical flow perceived by the driver as

4 $$\begin{aligned} u = - \left( \gamma + {\dot{\beta }} + {\dot{\phi }}_p \right) + \frac{V}{x_e^2 + y_e^2}\left( -x_e\sin \beta + y_e\cos \beta \right) - \frac{1}{x_e^2 + y_e^2}\left( \left( \gamma + {\dot{\beta }} + {\dot{\phi }}_p \right) \epsilon y_e + {\dot{\epsilon }} x_e \right) . \end{aligned}$$

(4) represents the optical flow of the target point $[x_e, y_e]^T$. We assume that the optical flow is generated on the ground ($z_e = 0$).

## Modeling of focus of expansion

One of the features of optical flow is the Focus of Expansion (FoE), which is the source point of the optical flow ($u = 0$ in (4)). Based on psychological experiments, the FoE is assumed to be capable of reflecting the curvilinear direction of self-motion of the vehicle’s movement (e.g.,^27,33^). When running in a straight line, the curvilinear direction of self-motion generated by FoE coincides with the linear instantaneous direction of travel. Whereas, during turning, the instantaneous direction of travel coincides with the tangent line of the curvilinear direction of self-motion. Thus, a driver can predict the future path of the vehicle from the optical flow according to the current vehicle state, and this information is useful for the steering performance required to track the target path. In this section, we derive three types of FoE: (1) the driver is seated at the center of the vehicle and gazes at the FP (Center-FP condition), (2) the driver is seated at either the left/right of the driver’s seat position and gazes at the FP (Seat-FP condition), (3) the driver is seated at either the left/right of the driver’s seat position and gazes at the TP (Seat-TP condition). These FoEs are useful in verifying the extent to which drivers can correctly perceive the future path of the vehicle.

### Focus of expansion in center-FP condition

In this subsection, we derive the FoE as the baseline condition when the driver is seated at the center of the vehicle $(\epsilon = {\dot{\epsilon }} = 0)$ and gazes at the FP. It is known from mathematical models and experimental results that the eye movement is about half of the vehicle yaw rate when the vehicle is traveling in a steady curve^18,19,31^. Thus, the eye movement referring to^31^ can be approximated as

5 $$\begin{aligned} {\dot{\phi }}_p = -\frac{1}{2}\gamma - {\dot{\beta }}. \end{aligned}$$

where this eye movement is zero if we assume that the drivers gaze at the TP since the TP fixation is not the waypoint strategy.

Even if the vehicle has a small lateral deviation from the target path, the eye movement during driving in the steady circular orbit can be assumed by (5). Substituting (5) into (4), the optical flow in this situation can be expressed as

6 $$\begin{aligned} u = -\frac{\gamma }{2} + \frac{V}{x_e^2 + y_e^2}\left( -x_e\sin \beta + y_e\cos \beta \right) . \end{aligned}$$

The FoE $(u = 0)$ in this situation is derived as

7 $$\begin{aligned} \left( x_e + \frac{V}{\gamma }\sin \beta \right) ^2 + \left( y_e - \frac{V}{\gamma }\cos \beta \right) ^2 = \left( \frac{V}{\gamma } \right) ^2. \end{aligned}$$

(7) can strictly reflect the vehicle motion while turning along the circular orbit. This means that the previous experimental results that the optical flow presents drivers with the future path of the vehicle is verified by theoretical optical flow theory. In addition, in the ideal state, the direction of self-motion of the vehicle perceived from optical flow is accurate no matter where drivers gaze on the ideal future path. Therefore, the assumption that the direction of self-motion obtained from optical flow determines the gaze point is not influenced by individual differences such as the gaze distance.

### Focus of expansion in seat-FP condition

Next, we assume a situation where the driver is seated in the driver’s seat position. In the previous subsection, the eye movement ${\dot{\phi }}_p$ when drivers gaze at the FP was mentioned. Similar to this, when the lateral deviation by the seat position is small, the eye movement ${\dot{\phi }}_p$ in this subsection is the same as in (5). The optical flow and FoE in this subsection can be represented as

8 $$\begin{aligned}&u = -\frac{\gamma }{2} + \frac{V}{x_e^2 + y_e^2}\left( -x_e\sin \beta + y_e\cos \beta \right) - \frac{1}{x_e^2 + y_e^2}\left( \frac{\gamma }{2}\epsilon y_e + {\dot{\epsilon }} x_e \right) , \end{aligned}$$

9 $$\begin{aligned}&x_e^2 + \left( y_e - \left( \frac{V}{\gamma } + \frac{\epsilon }{2} \right) \right) ^2 = \left( \frac{V}{\gamma } - \frac{\epsilon }{2} \right) ^2, \end{aligned}$$

where the lateral slip angle $\beta = {\dot{\beta }} = 0$ and ${\dot{\epsilon }} = 0$ in the FoE for simplicity.

### Focus of expansion in Seat-TP condition

Finally, we consider the situation where a driver with lateral deviation gazes at the TP. In the FP condition, the fixation distance is variable because the driver is assumed to gaze at the waypoint. However, the fixation distance can be assumed to be constant when they gaze at the TP in the circular path. Therefore, the lateral eye movement is not generated in the TP fixation condition. This is also verified by the experimental results^18,19^. Thus, the eye movement ${\dot{\phi }}_p$ is 0, and the optical flow and FoE that the driver perceive in this situation can be described as

10 $$\begin{aligned}&u = - \left( \gamma + {\dot{\beta }} \right) + \frac{V}{x_e^2 + y_e^2}\left( -x_e\sin \beta + y_e\cos \beta \right) - \frac{1}{x_e^2 + y_e^2}\left( \left( \gamma + {\dot{\beta }} \right) \epsilon y_e + {\dot{\epsilon }} x_e \right) , \end{aligned}$$

11 $$\begin{aligned}&x_e^2 + \left( y_e - \left( \frac{V}{2\gamma } + \frac{\epsilon }{2} \right) \right) ^2 = \left( \frac{V}{2\gamma } - \frac{\epsilon }{2} \right) ^2. \end{aligned}$$

## Comparison of drivers’ gaze based on focus of expansion

In this section, we compare the three types of FoE to interpret the difference between drivers’ gaze in the TP and FP fixations. We assume that drivers’ gaze can be interpreted in terms of the extent to which it is easy to perceive the future path of the vehicle. Ideally, the driver, who is seated at the vehicle center, gazes at the FP (Center-FP condition) and uses information around the FP for steering control. Then, the precise tracking control, wherein the target path and vehicle path (or FoE) are matched, can be achieved, because the FoE derived in (7) can strictly reflect the predicted vehicle path. However, the FoEs that the driver perceives in the real world are the Seat-FP or Seat-TP conditions as shown in (9) and (11). Drivers cannot correctly predict the vehicle motion because the FoEs they can perceive are inconsistent with the vehicle future path. Therefore, if the driver has a position bias such as Seat conditions, there is a possibility that gazing at the TP rather than the FP will be more effective for steering.

**Figure 4:**
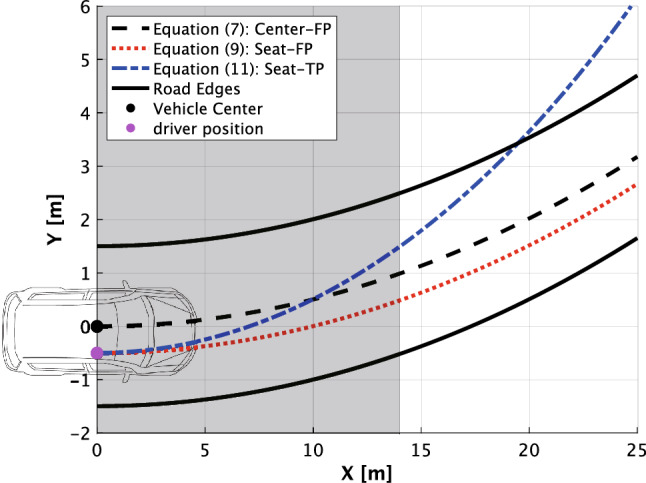
Comparison of the FoEs. [0, 0] is the position of the center of the vehicle. $[0, -0.5]$ is the driver’s head position. The black, red, and blue dotted lines represent the FoEs by (7), (9), and (11), respectively. A gray zone denotes that TP gaze is recommended because the distance between the black and blue dotted lines is smaller than the distance between the black and red dotted lines.

For instance, we consider a situation where the seat position (right side steering wheel) is 50 cm from the vehicle center, and the vehicle turns a counterclockwise curve with a radius of 100 m. In Fig. 4, the black dotted line denotes the FoE by (7) in the Center-FP condition, the red dotted line denotes the FoE by (9) in the Seat-FP condition, and the blue dotted line denotes the FoE by (11) in the Seat-TP condition. In this situation, the TP fixation is recommended in the area of [$$x < 14$$ m] for perceiving the correct future path because the difference between the black and blue dotted lines is smaller than that between the black and red dotted lines. On the other hand, the FP fixation is recommended at [$$ x > 14$$ m], as the red dotted line is close to the black dotted line. We consider that this difference between the FoEs in terms of perceiving the future path of the vehicle is important if drivers’ gaze strategy depends on optical flow.

**Figure 5:**
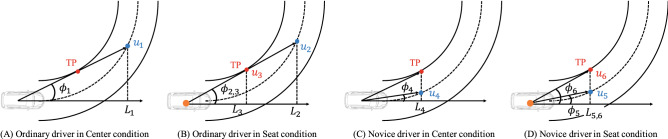
Definition of fixation points of ordinary and novice drivers.

In this study, we considered several situations in the numerical simulation to determine which gaze strategy is more effective in perceiving the vehicle future path. We ignored the influence of driver’s roll movement $({\dot{\epsilon }}_r = 0)$, and the seat position was only considered as the driver’s lateral deviation in this simulation. As mentioned in the introduction, in the concept of FP fixation, the gaze distance depends on the individual driver; thus, in this simulation, two types of gaze distances were assumed. Figure 5 shows the gaze strategies of ordinary and novice drivers. In the gaze strategy of an ordinary driver, shown in Fig. 5A,B, the FP was set on the target path beyond the TP by referring to^18,19^. The gaze distance of the FP in Fig. 5C,D was set to the same distance as the TP because novice drivers tend to gaze at the near region^34^. This difference between the gaze distance to the FP of the ordinary and novice drivers was defined in this paper as the difference in drivers’ skill levels. In addition, this difference between gaze points is also attributed to the difference in road conditions: an open curve for the ordinary driver and a closed curve for the novice driver. In the open curve environment, the driver can clearly see the entire view beyond the curve, whereas in the closed curve environment, the driver cannot predict the future orbit due to the presence of walls and other obstructions. In situations such as the closed curve, which restrict the driver’s view, the driver’s gaze distance is closer to him/her^35,36^. In these simulations, the ideal path for the individual driver was assumed to be the center of the road.

The optical flow derived in (6) in the Center-FP condition, which can reflect the restriction on vehicle motion, was denoted as $u_1, u_4$ in Fig. 5. They represent the positions where the driver gazes at far and near points on the target path. In the Seat-FP condition, the optical flow is represented as $u_2, u_5$ from (8), while that in the Seat-TP condition is indicated as $u_3, u_6$ from (10). (12) includes the eye movement condition derived in (5); that is, it expresses the optical flow that drivers perceive while turning along the steady circular path.

12 $$\begin{aligned} u_{ci} = \left\{ \begin{array}{lc} -\frac{\gamma }{2} + \frac{V}{L_i}\sin \phi _i\cos \phi _i &{} (i = 1, 4)\\ -\frac{\gamma }{2} + \frac{1}{L_i}\left( V - \frac{\epsilon \gamma }{2} \right) \sin \phi _i\cos \phi _i &{} (i = 2, 5)\\ -\gamma + \frac{1}{L_i}\left( V - \epsilon \gamma \right) \sin \phi _i\cos \phi _i &{} (i = 3, 6) \end{array} \right. , \end{aligned}$$

where we assume $\beta = {\dot{\beta }} = 0$ and $[x_e, y_e]^T = [L_i, L_i\tan \phi _i]^T$. $L_3 = L_5 = L_6$, $\phi _2 = \phi _3 = \phi _6$, and $u_3 = u_6$ are obvious from Fig. 5.

When we cannot assume the situation of the steady circular orbit, the eye movement should be sequentially calculated in the simulation. Then, the general optical flow is defined as

13 $$\begin{aligned} u_{gi} = \left\{ \begin{array}{ll} -\left( \gamma + {\dot{\phi }}_p \right) + \frac{V}{L_i}\sin \phi _i\cos \phi _i &{} (i = 1, 4)\\ -\left( \gamma + {\dot{\phi }}_p \right) + \frac{1}{L_i}\left( V - \epsilon \left( \gamma + {\dot{\phi }}_p \right) \right) \sin \phi _i\cos \phi _i &{} (i = 2, 3, 5, 6) \\ \end{array}. \right. \end{aligned}$$

Here, we define the two types of evaluation functions for ordinary and novice drivers respectively as

14 $$\begin{aligned} J_o= & {} \left( u_1 - u_3 \right) ^2 - \left( u_1 - u_2 \right) ^2, \end{aligned}$$

15 $$\begin{aligned} J_n= & {} \left( u_4 - u_6 \right) ^2 - \left( u_4 - u_5 \right) ^2. \end{aligned}$$

The optical flow shown by (12) and (13) can be regarded as the distance between an arbitrary point $[x_e, y_e]^T$ and the closest point on the FoE. Thus, the optical flow $u_1, u_4$, which can reflect the vehicle motion, and $u_2, u_3, u_5, u_6$, which the driver can perceive, are compared. The optical flow with the smaller difference from $u_1, u_4$ is effective in terms of perceiving the actual future path of the vehicle. The extent to which the driver can perceive the future path affects the accuracy of their steering performance. Therefore, the sign of the evaluation function can indicate the fixation strategy that is effective for the driver, as follows:

16 $$\begin{aligned} Estimated\ Gaze\ Strategy \left\{ \begin{array}{ll} Future\ Path\ Point &{} (J \ge 0)\\ Tangent\ Point &{} (J < 0) \end{array}. \right. \end{aligned}$$

## Numerical simulations

**Figure 6:**
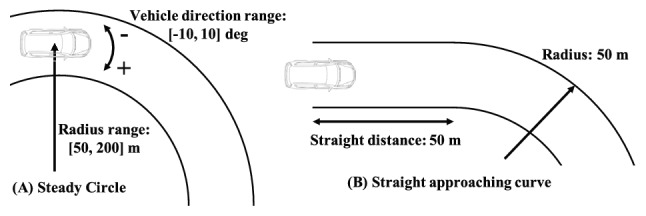
Simulation conditions. (**A**) and (**B**) show environments of a steady circular path and a straight path approaching a steady curve, respectively. The range of the vehicle direction is $[-10, 10]$ deg, and the radius of the path is [50, 200] m in steady circular path condition. The figure is taken from^29^.

In this section, we visualized the estimated gaze strategy based on optical flow theory through numerical simulations. Figure 6 shows the two types of driving conditions. (12) and (13) were used to evaluate the situations depicted in Fig. 6A,B, respectively. In all simulations, it was assumed that there was a right curve, the steering wheel was on the right side (0.35 m from the center in the car), and the velocity was 60 km/h.

### Simulation results for steady circle

**Figure 7:**
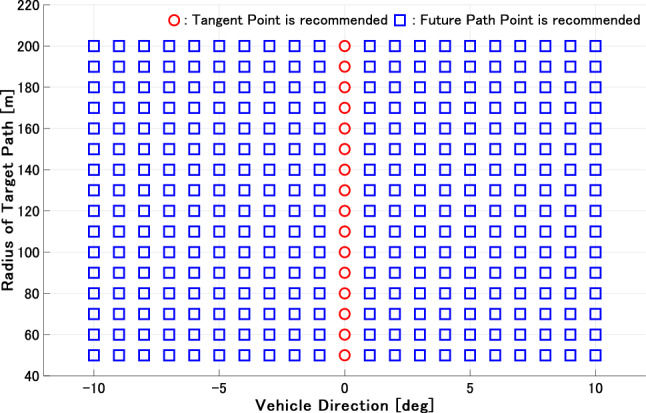
Simulation results of ordinary drivers on a curve. The red circle and blue square denote that the TP and FP fixation is recommended by the estimation of (14) and (16). The figure is taken from^29^.

**Figure 8:**
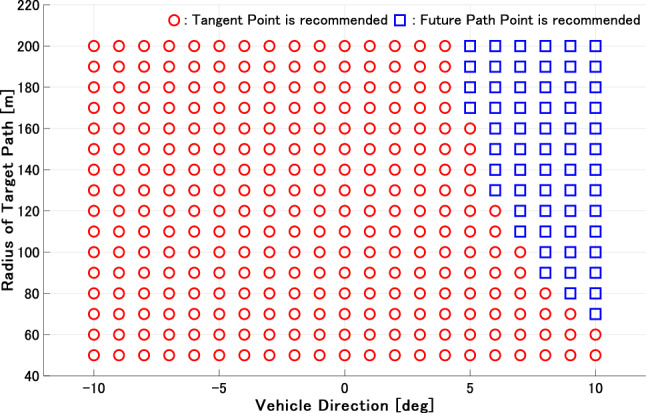
Simulation results of novice drivers on a curve. The red circle and blue square denote that the TP and FP fixation is recommended by the estimation of (15) and (16). The figure is taken from^29^.

Figures 7 and 8 show the results for the steady circle in each vehicle direction and the radius of the path. Figure 7 shows the estimated gaze strategy for an ordinary driver. The FP fixation is recommended based on the result under most conditions. The TP fixation is recommended to perceive the future path correctly only when the vehicle angle is 0 deg. This appears to be because the TP and FoE derived in (11), which the driver can perceive, are overlapped when the vehicle angle is 0 deg. Therefore, only this condition becomes the specific point where the TP is effective, as the optical flow $u_3$ on the TP is approximately 0 and simultaneously, $u_1 = 0$. On the other hand, Fig. 8 shows the estimated gaze strategy for a novice driver who gazes at the nearer area. The result demonstrates that the TP fixation is recommended to perceive the correct future path of the vehicle in the most area . In general, the driving experience affects the difference in gaze distance^34,37^. The results in this section have the potential to describe how driver behavior with regard to gaze distance affects the selection of gaze strategy.

We compare the results in this section to the results described in the literature. Previous studies^10^ claim that TP fixation is common, while other studies^11,16–19^ have shown drivers’ gaze at the FP rather than TP fixation. Studies^11,16^ have conducted simulator experiments to measure drivers’ gaze, where the drivers were seated at the center of the vehicle. Therefore, the results showing that drivers’ gaze is at the FP are obvious from optical flow theory. Studies^17–19^ conducting vehicle experiments have reported drivers’ gaze at the FP beyond the TP. These results are consistent with the results in this section. However^10^, demonstrated that drivers’ gaze accumulates in the TP as the path becomes a closed curve. This result corroborates the result in Fig. 8. Therefore, a driver’s fixation strategy may depend on optical flow, as the results in Figs. 7 and 8 are consistent with the results in the literature.

### Simulation results in straight path approaching a curve

Land^3,7^ first proposed the TP strategy and showed the drivers’ gaze at the TP before entering the curve. In the previous subsection, we discussed gaze strategy in a situation where there is no curvature change, such as the steady circle. In contrast, the TP fixation may be effective even for ordinary drivers when there is a curvature change.

**Figure 9:**
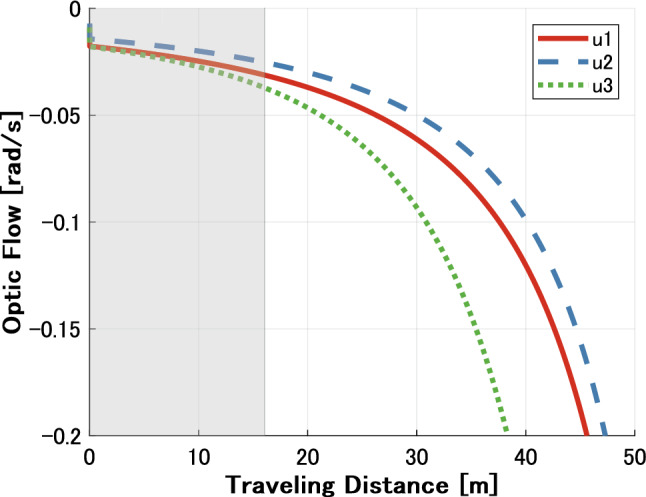
Simulation results of ordinary drivers while driving along a straight path approaching a circular path. The optical flows according to each situation in Fig. 5 represent $u_1$, $u_2$, and $u_3$ calculated by (6), (8), and (10). The gray zone denotes that the TP is recommended because the values of $u_1$ and $u_3$ are closer than the values of $u_1$ and $u_2$.

We compared the gaze strategy of ordinary drivers in a straight path approaching a curve, as shown in Fig. 6B. The eye movement ${\dot{\phi }}_p$ is sequentially calculated using (13). The experiments in the study by^3^ were conducted by an expert driver; therefore, we compared $u_1$, $u_2$, and $u_3$ to verify the gaze strategy for non-novice drivers. Figure 9 shows the results of comparison of the optical flow values. From the results, the TP fixation is recommended in the early stage of the straight path because the values of $u_1$ and $u_3$ are close, whereas the FP fixation is recommended after traveling approximately 16 m. This implies that the estimated gaze strategy may depend on the driving environment regardless of driving skill level. In addition, 1–2 s before entering the curve, expert drivers gaze at the TP in the reference Land^3^, whereas the TP estimated area in this study is over 2 s before entering the curve. This difference appears to be due to the velocity, curvature, and ideal path setting. Consequently, when there is a curvature change, there is also potential for the driver to gaze often at the TP.

## Experiments

In the previous section, the results of numerical simulations showed the potential for drivers’ gaze strategy to be interpreted as optical flow theory, which is the extent to which drivers can perceive the direction of self-motion. Therefore, in this section, experiments are conducted to compare driver gaze behavior to the gaze strategy generated by optical flow theory represented by (13). This experiment was approved by the Research Ethics Committee at Ritsumeikan University (approval number: Kinugasa-Hito-2018-71), and complied with all guidelines as set out in the declaration of Helsinki.

### Participants

An experiment was conducted with 10 university students (21–24 years old, mean age 22.75 years, mean years of licensure 2.92 years, ratio of male to female 9:1). All participants had normal vision and had held a driver’s license for at least one year. All participants signed informed consent, allowing for the collected data to be used for scientific purposes and publication. Participants received 1000JPY for their participation. Due to the low acquisition rate of two participants’ gaze data, their data were excluded from the analysis.

### Experimental environment

**Figure 10:**
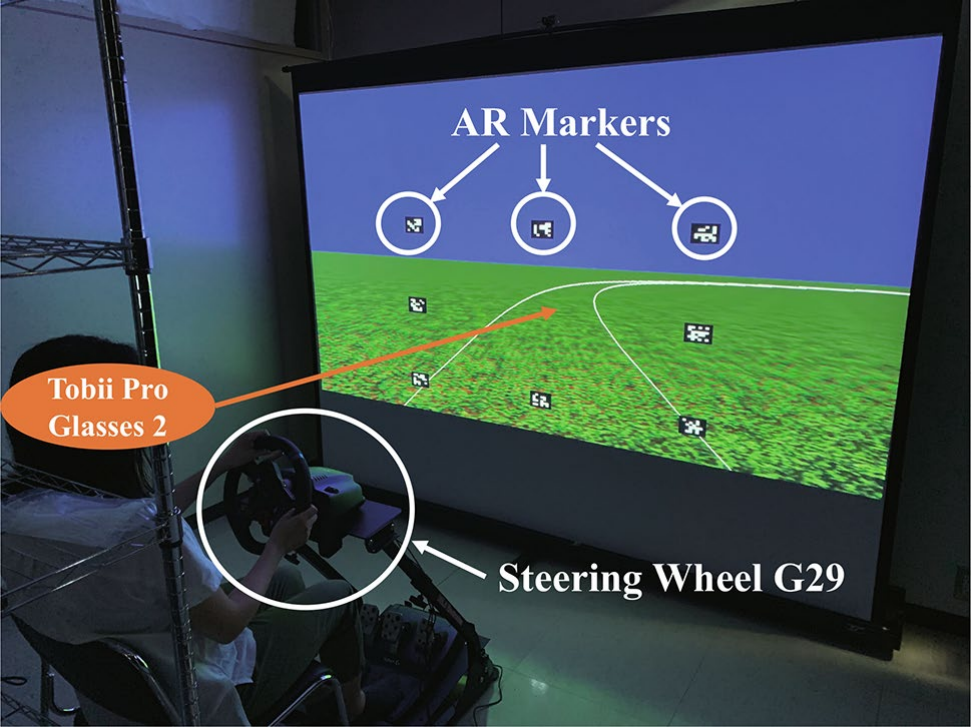
Driving simulator. Eight augmented reality (AR) markers were set on the screen to calculate drivers’ fixation.

The driving simulator used in our experiment is as shown in Fig. 10. The virtual environments were generated using Vizard 5.0 (WorldViz) and projected (BenQ TH671ST) at a size of $2.435 \times 1.38$ m; participants sat 1.4 m from the screen; and the eye-height was 1.5 m; thus, the field of view was $92.6 \times 52.5$ deg. The steering wheel was controlled using the Logitech G29 wheel (Logitech). The simulation was run in 40 Hz. To collect gaze data, participants wore Tobii Pro Glasses 2 (Tobii Technology K. K.) sampled in 50 Hz. We measured the gaze point in the virtual environments with two stages: 1. the gaze point of the driver on the screen (DriverToScreen), and 2. where the gaze point on the screen is projected in the virtual environment (ScreenToVirtual). In DriverToScreen, we used Tobii Pro Glasses 2 and AR markers on the screen. The calibration method and precision of Tobii Pro Glasses 2 are described in the official document^38^. The method of ScreenToVirtual is based on the Vizard library. Since the virtual environments are projected onto the screen using the geometric information of the real world such as the distance from the driver, the gaze point in the virtual environments can be calculated.

### Experimental design

**Figure 11:**
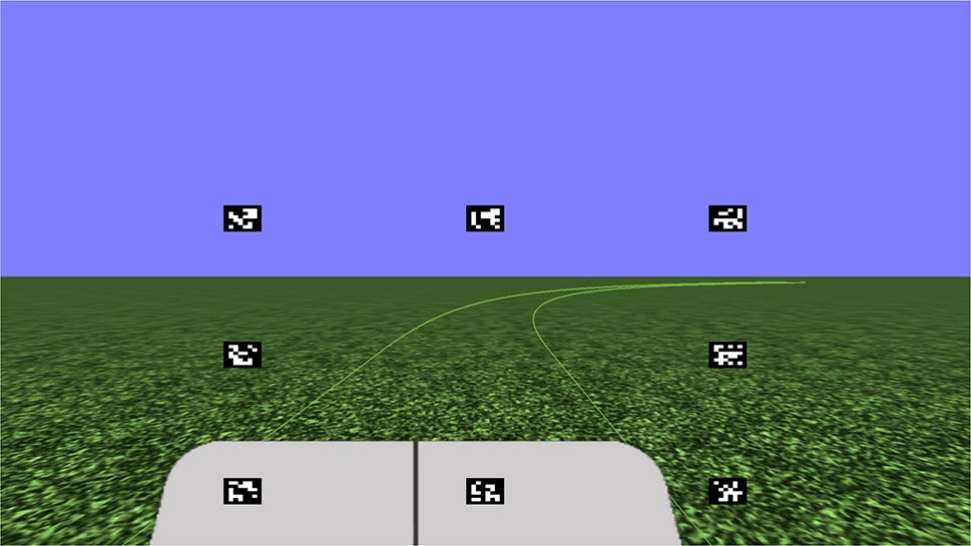
Simulation display. The gray zone imitates the vehicle body, and the black line in the grey zone shows the center of the vehicle. The driver position is on the right side of the vehicle.

Eight experimental conditions were applied: road width (3 and 5 m), vehicle speed (20 and 40 km/h), and road shape (circular and straight path). A road was generated with a combined steady circle of radius 80 m after a straight section. Two straight sections, of 10 and 40 m, were used for the circular and straight paths, respectively. Each condition had four trials (two left and two right turns); thus, a total of 32 runs were performed at random. Each trial lasted just 13 seconds, and as soon as one trial was over, the next began. Each trial was set to 13 s long because the vehicle finished driving the preparation course in about 15 s long. Previous psychological experiments have shown that driver behavior can be verified in a uniform environment, such as a simple circle, even for similarly short seconds. For instance, 6 s long for one trial was used in^36^. Therefore, drivers’ gaze behavior for 13 s long is enough to validate our hypothesis. All experiments were conducted with a right-hand drive (0.35 m from the center of the vehicle). As shown in Fig. 11, the center of the vehicle was displayed on the simulation screen, and to reduce the effect of individual differences, the participants were instructed to drive the vehicle so that its center overlapped the center of the road. To familiarize themselves with the driving simulator, participants were allowed to practice driving for approximately 5 mins before the experiment.

### Analysis

AOI (Area of Interest) and OKN (Optokinetic Nystagmus) methods are often used to analyze drivers’ gaze points^5^. However, in the present study, only the gaze points in the 3D simulation space were used for the analysis because the gaze points in the 3D environment could be measured accurately by the AR markers on the screen. We calculated the gaze point, TP, and FP in the 3D simulation environment for each frame. There is an infinite number of points on the future path, and the gaze distance depends on the individual drivers. In numerical simulations, the gaze distance was determined under the assumption of ordinary and novice drivers, but in experiments, the gaze distance should be selected for each driver. For this reason, the point on the road center close to the measured gaze point of the driver in each frame was set as the FP. The distances between the gaze point and TP/FP were calculated, and either the TP/FP closest to the gaze point was considered to comprise the observed driver’s gaze behavior. In addition, the calculated TP and FP, according to the current vehicle states, were used to calculate the gaze strategy based on optical flow theory. To exclude distraction situations, we excluded from the data instances when drivers gazed around AR markers and at points that exceeded 1.5 times the width of the road. A total of 122,736 valid data were gathered for the analysis, accounting for 90.3% of the total data.

### Experimental results

Table 1 shows the average rate of the observed drivers’ FP or TP fixations for each condition. The results showed no large difference based on road width or speed conditions, and the drivers often gazed at the FP. This result is consistent with the results of recent studies^4,11–19^. From the results in Table 1, detailed analyses under each condition, i.e., drivers’ gaze estimation by optical flow theory, will be discussed using the representative data.

**Table 1:** Results of average rate of observed drivers’ FP and TP fixations for each condition.

Path	Circular				Straight			
Velocity	20 km/h		40 km/h		20 km/h		40 km/h	
Road Width	3 m	5 m	3 m	5 m	3 m	5 m	3 m	5 m
FP Fixation Rate	97.4%	92.3%	95.8%	93.8%	98.2%	95.6%	95.5%	95.2%
TP Fixation Rate	2.6%	7.7%	4.2%	6.2%	1.8%	4.4%	4.5%	4.8%

Next, Table 2 shows the matching rate between the observed drivers’ gaze point and the calculated gaze strategy based on optical flow theory in circular and straight paths. Table 2 shows that, on average, the estimation of the gaze strategy by optical flow theory is 70.8% for the circular path and 65.1% for the straight path. There was some variability among participants, with 84.5% for those with high estimates and 58.3% for those with low estimates on the circular path, and 72.9% for those with high estimates and 56.4% for those with low estimates on the straight path. These results had a higher accuracy than the random estimation (50%). The estimation results were better for the circular path condition than for the straight path condition. This could be because the drivers’ gaze strategy was stable; thus, the gaze strategy was easily estimated under the circular condition.

**Table 2:** Results of matching rate between observed driver gaze point and theoretical gaze point based on optical flow theory for each participant.

Participant	Matching Rates	
Number	Circular Path	Straight Path
1	64.1%	63.6%
2	75.4%	69.5%
3	77.8%	65.6%
4	84.5%	70.3%
5	58.3%	56.4%
6	66.7%	59.6%
7	61.6%	63.3%
8	76.2%	72.9%
Average	70.8%	65.1%

**Figure 12:**
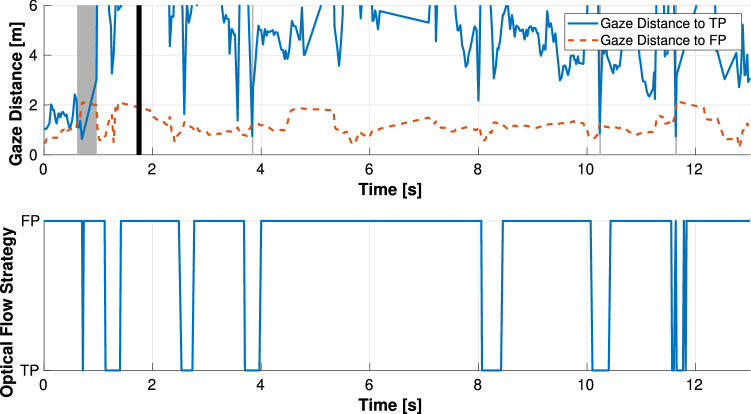
Result in curved situation. The top figure shows the gaze distance to TP/FP of the observed driver behavior. The gray zone means TP fixation strategy, while the black line is the boundary between the straight and curved sections. The bottom figure shows the calculated gaze strategy based on optical flow theory. The result estimates either FP or TP fixation behavior.

**Figure 13:**
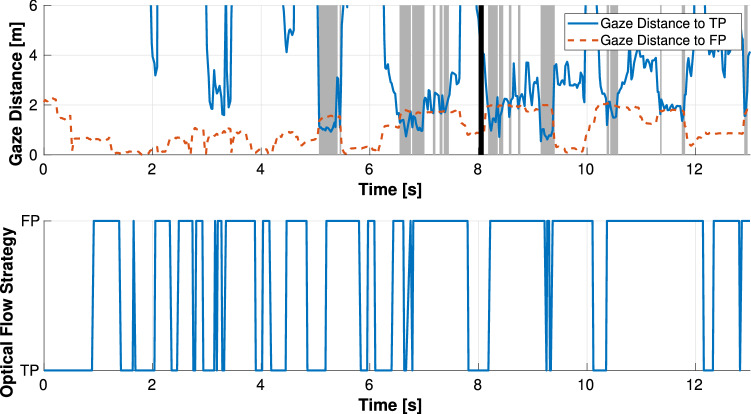
Result in straight situation. The top figure shows the gaze distance to TP/FP of the observed driver behavior. The gray zone means the TP fixation strategy, while the black line is the boundary between the straight and curved sections. The bottom figure shows the calculated gaze strategy based on optical flow theory. The result estimates either FP or TP fixation behavior.

Figures 12 and 13 show an example of a time plot of the observed gaze behavior compared to the calculated gaze strategy by optical flow theory in the circular and straight path conditions. From the results in the circular path shown in Figs. 12, drivers gazed at the FP most of the time while driving, and the calculated gaze strategy could estimate drivers’ gaze behavior because the TP fixation could be estimated at approximately 1, 4, 10, and 12 s. The calculated gaze strategy in the time after 2 and 8 s estimated the TP fixation, but the observed driver gaze strategy was FP fixation. However, at those times, the gaze distance to the TP was close to the gaze distance to the FP; thus, the calculated gaze strategy tended to estimate the observed behavior. On the other hand, for the results in the straight path shown in Fig. 13, the driver gazed more at the TP in the vicinity of where the straight and curved paths switched, whereas the driver gazed at the FP in the other regions. The observed TP fixation was not estimated accurately, particularly in the straight section.

While these results are from examples of many experimental data, others are similar to the data shown. The results demonstrate that optical flow theory can estimate drivers’ gaze strategy in a circular path more accurately than in a straight path. The reason for lower accuracy in estimating drivers’ gaze behavior in the straight path is likely because optical flow theory focuses only on the extent to which drivers can perceive the direction of self-motion. For instance, when the lateral deviation of the vehicle increases, it is clear that control for lane-keep becomes more important than predicting the future path of the vehicle. On the circular path, the vehicle control tends to be stable; therefore, it is important for the driver to predict the future path of the vehicle. On the other hand, in the vicinity of where the straight and circular paths are switched, the vehicle control tends to become unstable due to changes in curvature. In this case, the proposed estimation method could not be performed and the estimation accuracy was reduced.

According to the results of this study, the fact that it was possible to estimate 70.8% for the circular path and 65.1% for the straight path, even using only optical flow theory, is important for interpreting driver gaze behavior. To date, the reason why drivers gaze at the FP or TP has not been fully understood; however, the results of this study demonstrate that drivers’ gaze behavior might be determined by optical flow theory, which is the extent to which drivers can predict the future path of the vehicle. In addition to the results of this study, by combining road information with optical flow theory, we can estimate drivers’ gaze behavior more accurately since such behavior seems to also be determined by lane-keeping control. Consequently, we consider that the results of this study can contribute to future research in terms of elucidating holistic driver behavior including gaze strategy. Figuring out the drivers’ behavior can be useful for designing a driver-oriented autonomous vehicle that does not cause driver discomfort and for designing a Human-machine interface in harmony with the driver.

### Limitations

Finally, some limitations of this study are listed below.

- The results of this study show that the driver’s gaze point may be determined by the optical flow, but our hypothesis ignores many other environmental factors. Optical flow is just one aspect of how the driver’s gaze is determined.
- The optical flow theory we used for drivers’ gaze evaluation always assumes an ideal state. For instance, the drivers perceive optical flow generated on only the ground, and the lateral slip angle of the vehicle is ignored. Therefore, it is not an effective method for estimating the driver’s gaze point in actual vehicle experiments. However, this study does show the contribution as a fundamental study.
- Since the number of data per participant was large, the theory used in this study was validated by a sufficient number of data (122,735 situations in total). However, the sample size of the participants in the experiment is not large and therefore cannot be a general method of interpreting whole drivers’ gaze strategies.
- In this study, we discussed the driver’s gaze strategy as behavior for a single purpose, such as steering control. However, the driver’s gaze behavior is not performed for a single purpose. For example, look-ahead fixation, which is a gaze at more distant portions of the road, is performed for path planning^39^, and distraction to pay attention to traffic lights and pedestrians also affects the gaze behavior^40^. In addition, the driver does not always get information from the fovea, but can also get information from the peripheral vision^41^. The gaze strategy of the driver discussed in this study is not capable to explain these complex gaze behaviors.

## Conclusion

This study aimed to interpret the difference between the Tangent Point (TP) and Future Path Point (FP) as types of drivers’ gaze behavior. Most previous studies have demonstrated that drivers gaze at the FP rather than the TP, but the reason for their gaze strategy, i.e., why they gaze at these points while driving, has not been fully understood. In this study, we hypothesized that drivers’ gaze strategy is determined by optical flow. We modeled the optical flow and quantified the extent to which drivers can perceive the predicted future path of the vehicle using optical flow. The results of numerical simulations demonstrated that optical flow theory has the potential to reflect drivers’ gaze behavior in both circular and straight path situations by comparing our results with previous findings from the literature^3,10,11,16–19^. In the experiment, the observed drivers’ gaze behavior was compared to the estimated drivers’ gaze strategy generated by optical flow theory. The results demonstrated that the observed drivers’ gaze behavior could be estimated with an accuracy of 70.8% and 65.1% in the circular and straight paths, respectively. The estimation in the circular path was higher than that in the straight path, probably because vehicle control was more stable on the circular path. Consequently, the study results suggest that optical flow theory could be a determining factor in drivers’ gaze strategy.

However, while we hypothesized that drivers’ gaze behavior is influenced by optical flow, drivers’ gaze is obviously determined by many other factors, including the road information. Therefore, while the results of this study are a factor in understanding drivers’ gaze behavior, drivers’ gaze behavior needs to be discussed from a more multifaceted perspective in order to fully understand it.

## Data Availability

The datasets generated and/or analyzed during the current study are available from the corresponding author on reasonable request.

## References

[1] Land MF (2006). Eye movements and the control of actions in everyday life. Retin. Eye Res..

[2] Lappi O, Mole CD (2018). Visuomotor control, eye movements, and steering: A unified approach for incorporating feedback, feedforward and internal models. Psychol. Bull..

[3] Land MF, Lee DN (1994). Where we look when we steer. Nature.

[4] Tuhkanen S (2019). Humans use predictive gaze strategies to target waypoints for steering. Sci. Rep..

[5] Lappi O (2014). Future path and tangent point models in the visual control of locomotion in curve driving. J. Vis..

[6] Lappi O, Rinkkala P, Pellanen J (2014). Systematic observation of an expert driver’s gaze strategy—an on-road case study. Front. Psychol..

[7] Land MF, Tatler BW (2001). Steering with the head: The visual strategy of a racing driver. Curr. Biol..

[8] Mars F (2008). Driving around bends with manipulated eye-steering coordination. J. Vis..

[9] Kandil FI, Rotter A, Lappe M (2009). Driving is smoother and more stable when using the tangent point. J. Vis..

[10] Kandil FI, Rotter A, Lappe M (2010). Car drivers attend to different gaze targets when negotiating closed vs. open bends. J. Vis..

[11] Wilkie RM, Wann JP (2003). Eye-movements aid the control of locomotion. J. Vis..

[12] Wann JP, Swapp DK (2000). Why you should look where you are going. Nat. Neurosci..

[13] Robertshaw KD, Wilkie RM (2008). Does gaze influence steering around a bend?. J. Vis..

[14] Wilkie RM, Kountouriotis GK, Merat N, Wann JP (2010). Using vision to control locomotion: Looking where you want to go. Exp. Brain Res..

[15] Mars F, Navarro J (2012). Where we look when we drive with or without active steering wheel control. PLoS ONE.

[16] Kountouriotis GK, Floyd RC, Gardner PH, Merat N, Wilkie RM (2012). The role of gaze and road edge information during high-speed locomotion. J. Exp. Psychol. Hum. Percept. Perform..

[17] Lappi O, Lehtonen E, Pekkanen J, Itkonen T (2013). Beyond the tangent point: Gaze targets in naturalistic driving. J. Vis..

[18] Lappi O, Pekkanen J, Itkonen TH (2013). Pursuit eye-movements in curve driving differentiate between future path and tangent point models. PLoS ONE.

[19] Itkonen T, Pekkanen J, Lappi O (2015). Driver gaze behavior is different in normal curve driving and when looking at the tangent point. PLoS ONE.

[20] Ren, Y. Y., Li, X. S., Zheng, X. L., Li, Z. & Zhao, Q. C. Analysis of drivers’ eye-movement characteristics when driving around curves. *Hindawi* **462792**, 1–10 (2015).

[21] van Leeuwen PM, de Groot S, Happee R, de Winter JCF (2017). Differences between racing and non-racing drivers: A simulator study using eye-tracking. PLoS ONE.

[22] Vansteenkiste P (2014). Cycling around a curve: The effect of cycling speed on steering and gaze behavior. PLoS ONE.

[23] Psychol Traffic, Lobjois R, Siegler IA, Mars F (2016). Effects of visual roll on steering control and gaze behavior in a motorcycle simulator. Transp. Res. Part F Traffic Psychol. Behav..

[24] Land MF, Horwood J (1995). Which parts of the road guide steering?. Nature.

[25] Wilkie RM, Wan JP (2003). Controlling steering and judging heading: Retinal flow, visual direction, and extraretinal information. J. Exp. Psychol. Hum. Percept. Perform..

[26] Gibson JJ (1950). The Perception of the Visual World.

[27] Warren WH, Mestre DR, Blackwell AW, Morris MW (1991). Perception of circular heading from optical flow. J. Exp. Psychol. Hum. Percept. Perform..

[28] Okafuji, Y., Wada, T., Sugiura, T., Murakami, K. & Ishida, H. Drivers’ gaze behaviors are influenced by vehicle position. In *Proceedings of the Human Factors and Ergonomics Society Annual Meeting*, 2020).

[29] Okafuji, Y., Fukao, T. & Inou, H. Theoretical interpretation of driver’s gaze considering optic flow and seat position. In *Proceedings of 14th 14th IFAC Symposium on Analysis Design and Evaluation of Human Machine Systems*, Vol. 52, no. 19, 335–340 (2019).

[30] Gibson JJ (1958). Visually controlled locomotion and visual orientation in animals. Br. J. Psychol..

[31] Okafuji Y, Fukao T, Yokokohji Y, Inou H (2016). Design of a preview driver model based on optical flow. IEEE Trans. Intell. Veh..

[32] Schweigart G, Mergner T, Evdokimidis I, Morand S, Becker W (1997). Gaze stabilization by optokinetic reflex (OKR) and vestibulo-ocular reflex (VOR) during active head rotation in man. Vis. Res..

[33] Kim NG, Turvey MT (1998). Visually perceiving heading on circular and elliptical paths. J. Exp. Psychol. Hum. Percept. Peform..

[34] Mourant RR, Rockwell TH (1972). Strategies of visual search by novice and experienced drivers. Hum. Factors.

[35] Frissen I, Mars F (2014). The effect of visual degradation on anticipatory and compensatory steering control. Q. J. Exp. Psychol..

[36] Mole CD, Kountouriotis G, Billington J, Wilkie RM (2016). Optic flow speed modulates guidance level control: New insights into two-level steering. J. Exp. Psychol. Hum. Percept. Perform..

[37] van Leeuwen PM, Happee R, de Winter JCF (2015). Change of driving performance and gaze behavior of novice drivers during a 30-min simulator-based training. Procedia Manuf..

[38] *Eye tracker data quality report: Accuracy, precision and detected gaze under optimal conditions—controlled environment* https://bit.ly/36nSGqc (2017).

[39] Lehtonen E, Lappi O, Koirikivi I, Summala H (2014). Effect of driving experience on anticipatory look-ahead fixations in real curve driving. Accid. Anal. Prev..

[40] Kircher, K., Ahlstrom, C. & Kircher, A. Comparison of two eye-gaze based real-time driver distraction detection algorithms in a small-scale field operational test. In *Proceedings of the Fifth International Driving Symposium on Human Factors in Driver Assessment, Training, and Vehicle Design* 16–23 (2009).

[41] Lehtonen E (2018). Gaze doesn’t always lead steering. Accid. Anal. Prev..

